# Factors associated with pathologic myopia onset and progression: A systematic review and meta-analysis

**DOI:** 10.1111/opo.13312

**Published:** 2024-04-02

**Authors:** Fabian Yii, Linda Nguyen, Niall Strang, Miguel O. Bernabeu, Andrew J. Tatham, Tom MacGillivray, Baljean Dhillon

**Affiliations:** 1 Centre for Clinical Brain Sciences, The University of Edinburgh, Edinburgh, UK; 2 Curle Ophthalmology Laboratory, Institute for Regeneration and Repair, The University of Edinburgh, Edinburgh, UK; 3 MRC Human Genetics Unit, Institute of Genetics and Cancer, The University of Edinburgh, Edinburgh, UK; 4 Department of Vision Sciences, Glasgow Caledonian University, Glasgow, UK; 5 Centre for Medical Informatics, Usher Institute, The University of Edinburgh, Edinburgh, UK; 6 The Bayes Centre, The University of Edinburgh, Edinburgh, UK; 7 Princess Alexandra Eye Pavilion, NHS Lothian, Edinburgh, UK

**Keywords:** epidemiology, longitudinal, META-PM, pathologic myopia, prognostic factors, risk factors

## Abstract

**Purpose:**

To synthesise evidence across studies on factors associated with pathologic myopia (PM) onset and progression based on the META-analysis for Pathologic Myopia (META-PM) classification framework.

**Methods:**

Findings from six longitudinal studies (5–18 years) were narratively synthesised and meta-analysed, using odds ratio (OR) as the common measure of association. All studies adjusted for baseline myopia, age and sex at a minimum. The quality of evidence was rated using the Grades of Recommendation, Assessment, Development and Evaluation framework.

**Results:**

Five out of six studies were conducted in Asia. There was inconclusive evidence of an independent effect (or lack thereof) of ethnicity and sex on PM onset/progression. The odds of PM onset increased with greater axial length (pooled OR: 2.03; 95% CI: 1.71–2.40; *p* < 0.001), older age (pooled OR: 1.07; 1.05–1.09; *p* < 0.001) and more negative spherical equivalent refraction, SER (OR: 0.77; 0.68–0.87; *p* < 0.001), all of which were supported by an acceptable level of evidence. Fundus tessellation was found to independently increase the odds of PM onset in a population-based study (OR: 3.02; 2.58–3.53; *p* < 0.001), although this was only supported by weak evidence. There was acceptable evidence that greater axial length (pooled OR: 1.23; 1.09–1.39; *p* < 0.001), more negative SER (pooled OR: 0.87; 0.83–0.92; *p* < 0.001) and higher education level (pooled OR: 3.17; 1.36–7.35; *p* < 0.01) increased the odds of PM progression. Other baseline factors found to be associated with PM progression but currently supported by weak evidence included age (pooled OR: 1.01), severity of myopic maculopathy (OR: 3.61), intraocular pressure (OR: 1.62) and hypertension (OR: 0.21).

**Conclusions:**

Most PM risk/prognostic factors are not supported by an adequate evidence base at present (an indication that PM remains understudied). Current factors for which an acceptable level of evidence exists (limited in number) are unmodifiable in adults and lack personalised information. More longitudinal studies focusing on uncovering modifiable factors and imaging biomarkers are warranted.

**Supplementary Information:**

The online version of this article (doi:10.1111/opo.13312) contains supplementary material, which is available to authorized users.

## Key points

- While the mechanism and pathophysiology of pathologic myopia are relatively well covered in the existing literature, no work has systematically reviewed and critically appraised the evidence base for factors associated with its development and progression.
- Most reviewed factors, such as sex, ethnicity and baseline severity of myopic maculopathy, are not currently supported by an acceptable level of evidence—except for myopia severity (for both pathologic myopia onset and progression), age (onset) and education level (progression).
- More longitudinal studies are needed to uncover and corroborate the independent effect of modifiable health or lifestyle factors (as effective treatment is currently unavailable) and imaging biomarkers (to facilitate personalised risk prediction).

## INTRODUCTION

In one of their seminal works describing pathologic fundus changes secondary to excessive axial length, Curtin and Karlin^1^ noted that these changes ‘are as striking as they are unique’. Yet, it was not until 45 years later, in 2015, that an international panel of established myopia researchers and retinal specialists agreed upon a common classification framework for pathologic myopia (PM) based on colour fundus photographs (META-analysis for Pathologic Myopia or META-PM),^2^ which had hitherto been subjected to considerable inconsistencies in nomenclature. For instance, PM was often used interchangeably, but erroneously, with high myopia, with little consideration of whether complications characteristic of excessive axial length were evident.^3–5^

Under the META-PM framework, myopic maculopathy—a prominent feature of PM characterised by progressive macular degeneration—is categorised into normal, fundus tessellation, diffuse chorioretinal atrophy, patchy chorioretinal atrophy and macular atrophy.^2^ Three additional ‘plus’ lesions (lacquer cracks, myopic choroidal neovascularisation and Fuchs spot), so-called because they can develop from or coexist with any myopic maculopathy category, were also incorporated into META-PM.^2^ PM is defined as myopic maculopathy equal to or more serious than diffuse chorioretinal atrophy, or the presence of any ‘plus’ lesion(s) or posterior staphyloma.^2,6^

Driven by a high prevalence of myopia in East Asia,^7^ PM has long been a leading cause of visual impairment among adults in parts of Mainland China,^8^ Taiwan^9^ and Japan.^10,11^ In keeping with a projected steep rise in myopia prevalence worldwide (from around 23% in 2000 to 50% by 2050),^7^ the global prevalence of PM-induced visual impairment is predicted to increase from 0.13% in 2015 to 0.57% by 2050.^12^ In addition to substantial potential productivity loss (US$2 billion based on conservative estimates),^13^ PM reduces the quality of life—even when distance visual acuity is adjusted for, indicating that its impact stretches far beyond spatial resolution.^14,15^

The intervening years following Curtin and Karlin^1^ saw the introduction of various PM classification systems,^16–18^ but none were universally adopted. This precluded systematic comparison and integration of earlier research on factors associated with PM *onset* (*risk* factors) and *progression* (*prognostic* factors).^19–21^ In the light of this, we aimed to synthesise evidence across longitudinal studies that adopted META-PM to determine the *adjusted* risk estimates for various risk and prognostic factors; that is, risk estimates over and above the effect of a pre-defined set of core factors. Core factors included baseline age, sex and the baseline severity of myopia (in terms of axial length or refractive error). Table 1 presents the review objectives in the PICOTS (Population, Index factors, Comparator factors, Outcomes, Timing and Setting) format.^22,23^

**TABLE 1:** Review objectives in the PICOTS format.

PICOTS element	Description
Population	*Risk* factors: Individuals with any degree of myopia (spherical equivalent refraction ≤ −0.50 D) who do not have a diagnosis of PM at baseline per the META-PM definition *Prognostic* factors: Treatment-naive myopes (not being treated for PM), who already have a diagnosis of PM at baseline per the META-PM definition
Index factors	All types of factors are of interest, whether they are modifiable or unmodifiable, including demographic, behavioural, environmental and physiological factors
Comparator factors	Comparator (core) risk and prognostic factors include baseline age, sex and baseline severity of myopia (in terms of axial length or refractive error)
Outcomes	PM onset and PM progression
Timing	At least 1 year between baseline and follow-up. Risk and prognostic factors are measured around the same time as when the fundus image, on which PM grading is performed, is taken at both baseline and follow-up
Setting	Intended setting is primary care to aid risk stratification during routine eye examinations

Abbreviations: META-PM, META-analysis for Pathologic Myopia; PM, pathologic myopia.

## METHODS

The review protocol was registered prospectively on PROSPERO (CRD42022378743) before formal screening of studies began. The findings were reported in reference to the Preferred Reporting Items for Systematic Reviews and Meta-Analyses (PRISMA) guidelines.^24^

### Definition of PM onset and progression

We acknowledge that posterior staphyloma is best imaged with a widefield imaging modality because the predominant subtype of posterior staphyloma often extends beyond the field of view of a conventional fundus camera.^25^ As we anticipated few studies to have access to widefield imaging modalities—and the authors might therefore choose not to grade posterior staphyloma—we decided not to incorporate posterior staphyloma into our definition of PM. That said, studies incorporating posterior staphyloma would still be included, in which case they would be analysed separately. PM onset was defined as the development of myopic maculopathy equal to or more serious than diffuse chorioretinal atrophy—or any of the three ‘plus’ lesions—among myopes without PM at baseline; PM progression was defined as an increase in myopic maculopathy category (from diffuse chorioretinal atrophy at the very least), enlargement of existing chorioretinal atrophy, enlargement of existing ‘plus’ lesion(s) or the development of new ‘plus’ lesion(s) among myopes with a diagnosis of PM at baseline.

### Electronic literature searches

We searched MEDLINE (Ovid), EMBASE (Ovid) and Scopus from 1 January 2015 (the year META-PM was developed) to 27 November 2022 while restricting the searches to human studies and publications in English. We included the following search keywords related to PM (Medical Subject Headings, MeSH, indicated where appropriate), including their spelling and associated variants: ‘degenerative myopia’ (MeSH), ‘choroidal neovascularization’ (MeSH), ‘myopia’ (MeSH), ‘myopic maculopathy’, ‘myopic macular degeneration’, ‘staphyloma’, ‘lacquer crack’, ‘fuchs spot’, ‘tessellated fundus’, ‘tessellated retina’, ‘diffuse chorioretinal atrophy’, ‘patchy chorioretinal atrophy’, ‘macular atrophy’ and ‘pathologic myopia’.

In addition to these PM-specific search terms, we applied a validated search filter to limit the search results to prognostic research (Ingui filter),^26^ which included the following keywords: ‘validation’, ‘prediction’, ‘rule’, ‘outcome’, ‘risk’, ‘model’, ‘history’, ‘variable’, ‘criteria’, ‘score’, ‘characteristic’, ‘finding’, ‘factor’, ‘decision’ and ‘prognosis’. The full search strategy for each electronic database can be found in Appendix S1–S3.

### Inclusion and exclusion criteria

We included any longitudinal observational studies, irrespective of whether these were (prospective or retrospective) cohort or (nested or classic) case–control studies. Readers may refer to Biesheuvel et al.^27^ for a comparison between classic and nested case–control designs. Song and Chung^28^ on the other hand, provide a useful overview of the distinction between a retrospective cohort design and a case–control design. Studies that were purely descriptive (e.g., case series, case report), without full-text articles or not reported in English were excluded. We focused on individuals aged ≥7 years of age at baseline to reduce the likelihood of including patients with congenital or syndromic forms of myopia, such as myopia secondary to Marfan syndrome and Stickler syndrome. Myopia (spherical equivalent refraction, SER ≤ −0.50 D)^29^ of any severity was accepted because myopic pathology is not found exclusively in high myopes.^25^ We excluded studies involving participants who were receiving or had received treatment in relation to PM, such as anti-vascular endothelial growth factor (anti-VEGF) therapy for myopic choroidal neovascularisation.

We did not restrict the present review to factors of a particular type, whether these were modifiable or non-modifiable, nor did we exclude studies based on the method employed to measure a given factor, though the latter might have bearing on the risk of bias assessment detailed later. We excluded studies that analysed PM onset and PM progression as a single event. For inclusion, studies had to grade PM based on colour fundus photographs at a minimum and not be limited to the investigation of a limited subset of PM complications (e.g., considering only the ‘plus’ lesions). Studies that investigated factors associated with PM recurrence or treatment response were also excluded. Included studies were required to have a follow-up duration of at least 1 year.

### Study selection and data extraction

All references retrieved by electronic searches as detailed above were imported into Covidence (covidence.org). Following automatic deduplication, two reviewers (FY and LN) screened the imported abstracts independently and resolved any disagreements through discussion or involved a third reviewer if necessary. The reviewers then obtained the full-text copies of eligible abstracts and further assessed their eligibility independently, following the same process to resolve any disagreements. A data extraction form containing items, including study design, participant details, PM grading protocol, sample size, and unadjusted and adjusted effect measures, was created using Covidence. The two reviewers piloted the form on one study^30^ and made appropriate refinement to the form before proceeding further. They performed data extraction independently and followed the same process as above to resolve any disagreements.

### Assessment of risk of bias

We used the Quality in Prognosis Studies^31^ tool to assess the risk of bias arising from six different domains, each with its own set of signalling items (see Appendix S4). We gave a study an overall ‘low level of concern’ rating if all six domains were judged to have low risk, ‘moderate level of concern’ if no domain was judged to have high risk but at least one was judged to have moderate risk and ‘high level of concern’ if at least one domain was judged to have high risk. We adapted the ‘study confounding’ domain to ‘inappropriate adjustment for core factors’ to reflect our focus on the independent effect of each factor after adjusting for the pre-defined core factors. The same reviewers assessed each study independently and resolved any disagreements following the same process as above.

### Data synthesis

We used odds ratio (OR) in the natural log scale as the common measure of association for meta-analysis owing to its favourable mathematical properties (e.g., unbounded).^32,33^ When reporting or communicating effect, we transformed OR back to the original linear scale. Where studies reported risk ratio, conversion to OR was performed using the following equation^34,35^:

1 $$ \mathrm{Odds}\ \mathrm{ratio}=\frac{-\mathrm{risk}\ \mathrm{ratio}+\left(\mathrm{BR}\times \mathrm{risk}\ \mathrm{ratio}\right)}{\left(\mathrm{BR}\times \mathrm{risk}\ \mathrm{ratio}\right)-1}, $$

where BR refers to baseline risk and was assumed to be equal to the reported PM incidence rate or PM progression rate—whichever was applicable. The corresponding authors of included studies would be contacted to retrieve any relevant missing information. We had planned to perform meta-analyses when there were at least two studies homogeneous enough for the pooled effect measure to be meaningful. ‘Homogeneous’ was defined as having the same outcome definition (whether posterior staphyloma was included) and adjustment for core risk/prognostic factors. In our initial (a priori) definition of ‘homogeneous’, we additionally included similar follow-up duration (i.e., difference not larger than 1 year) as another criterion for meta-analysis because we anticipated the risk estimates to be systematically larger in longer term studies, given that more cases might develop or progress over the longer term. However, we observed a posteriori that the direction of association or effect size of a factor did not differ systematically based on follow-up duration, and therefore chose to meta-analyse studies irrespective of the differences in their follow-up length.

The OR was pooled for each risk factor and prognostic factor separately. The generic inverse-variance method^33^ was used to calculate the weighted average of OR. If there were five or more studies to be pooled, we would use a random-effects model^36^; otherwise, a fixed-effects model would be employed because an accurate estimate of between-studies variance cannot be obtained with a random-effects model when the number of studies is small.^37,38^ All analyses were carried out using the *metafor* package in R (v4.2.2; R Core Team 2022, r-project.org).

### Rating of quality of evidence

We used a modified version of the Grades of Recommendation, Assessment, Development and Evaluation framework^39^ to assess the overall quality of evidence for each risk and prognostic factor. The phase of investigation (exploratory or confirmatory),^40^ study limitations (risk of bias), inconsistency (direction of effect), indirectness (similarity between study sample and our targeted population), imprecision (spread of 95% CI), publication bias (whether the factor was underexplored), effect size (for categorical factors) and dose effect (for continuous factors) were considered in our assessment (detailed in Appendix S5). The findings of this review would be discussed in the light of the quality of evidence. FY carried out the assessment in consultation with BD.

## RESULTS

### Characteristics and risk of bias of included studies

**FIGURE 1:**
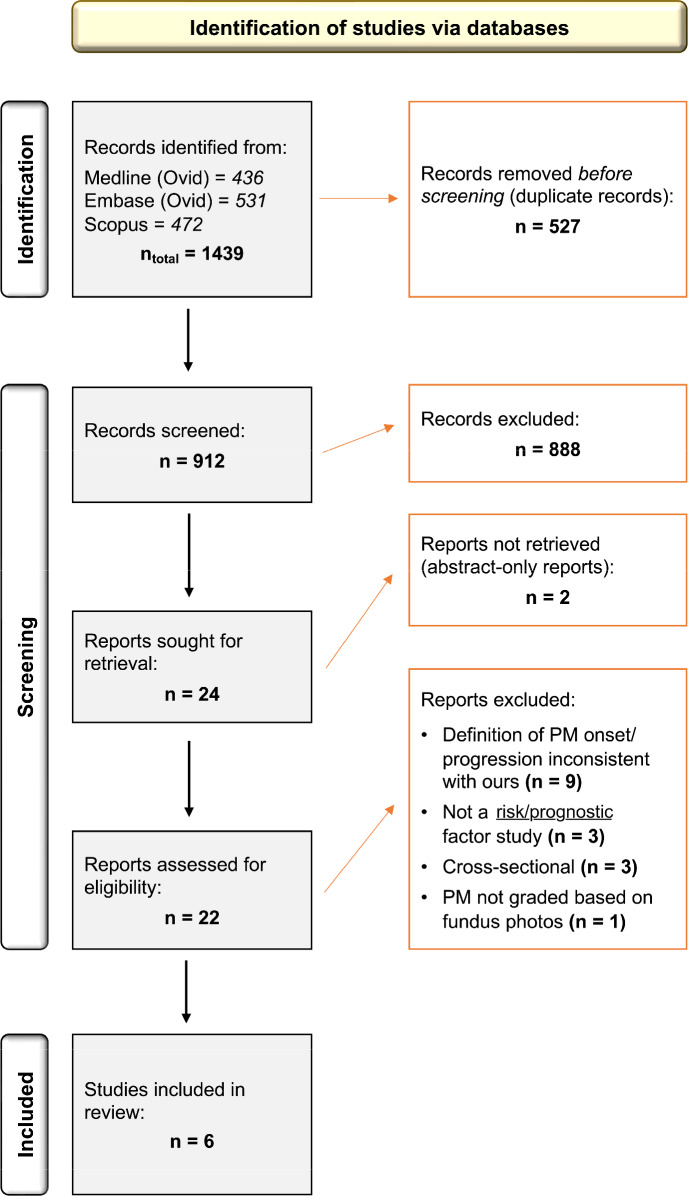
The Preferred Reporting Items for Systematic Reviews and Meta-Analyses flow diagram. Sixteen studies were excluded during full-text screening: nine were rejected because their definition of pathologic myopia (PM) onset/progression was not consistent with ours (e.g., PM onset and progression analysed as a single event); three were rejected because they looked only at the natural history of PM; three were rejected because they were cross-sectional studies; and one was rejected because PM was graded based only on widefield imaging.

Electronic searches identified 1439 references. After removing 527 duplicate records, 912 abstracts were screened. Of these, 22 studies entered full-text screening, after which six studies^30,41–45^ were finally included. More details are provided in the PRISMA flow diagram below (Figure 1). The inter-rater reliability (Cohen's Kappa) for abstract and full-text screening was 0.57 (moderate) and 0.80 (high), respectively.

Tables 2 and 3 provide a summary of the key characteristics and risk of bias for each study. Most studies^41–45^ employed a nested case–control design in that their cases and controls were derived from well-defined, population-based cohort studies. However, Fang et al.^30^ obtained their data from the medical records of a specialist high myopia clinic. We rated this study as having an overall moderate risk of bias because the study participants could not be assumed to be representative of the general population. Lin et al.,^43^ Hopf et al.^42^ and Ueda et al.^44^ worked with the data from the Handan Eye Study (5 years),^46^ Gutenberg Health Study (5 years)^47^ and Hisayama Study (5 years),^48,49^ respectively. Both Foo et al.^41^ (12 years) and Wong et al.^45^ (6 years) worked with the data from the multi-ethnic (comprising Malays, Indians and Chinese) Singapore Epidemiology of Eye Diseases (SEED) study,^50^ but the former did not include Chinese participants because they had yet to reach 12 years of follow-up at the time of the study. We judged the overall risk of bias of Foo et al.^41^ as high due to significant attrition (87.1%), while Ueda et al.^44^ and Hopf et al.^42^ as moderate on account of their relatively low participation rate at baseline (<67% but >50%).

**TABLE 2:** Key characteristics of included studies.

Study	Sample	Eyes	BL age (y)	BL SER (D)	BL AL (mm)	FU (y)	RF	PF
Foo et al. (2022)^41^	Malays & Indians	H: 1504 PM: 75	H: 51.9 ± 7.8 PM: 57.5 ± 8.7	H: −1.9 ± 2.1 PM: −6.4 ± 4.6	H: 24.1 ± 1.2 PM: 26.4 ± 2.2	12	Age, sex, AL, SER, ethnicity, edu, tessellated fundus	Age, sex, AL, SER, ethnicity, edu
Fang et al. (2018)^30^	Japanese	H: 289 PM: 521	H: 32.4 ± 16.5 PM: 47.7 ± 14.2	H + PM: −13.2 ± 4.8	H: 27.6 ± 1.4 PM: 29.4 ± 1.8	18	Age, sex, AL, change in AL, G/D, FU, MM	Age, sex, AL, change in AL, G/D, FU
Lin et al. (2018)^43^	Chinese	H: 2287 PM: 51	H: NA PM: 59.1 ± 13.2	H: NA PM: −11.6 (range: −0.8 to −26.5)	H: NA PM: 27.5 (range: 22.5 to 33.1)	5	Not tested: only 5 out of 2287 eyes developed PM	Age, sex, AL, SER, edu, hypertension, change in SER
Wong et al. (2020)^45^	Malays, Indians & Chinese	H: 3373 PM: 288	H + PM: 54.2 ± 8.4	H + PM: −2.9 ± 2.8	H + PM: 24.6 ± 1.5	6	Age, sex, AL, SER, ethnicity, edu, cataract	Age, sex, AL, SER, ethnicity, edu, MM
Ueda et al. (2020)^44^	Japanese	H: 2164 PM: 0	H: 62.4 ± 10.9 PM: NA	H: −0.7 ± 1.6 PM: NA	H: 23.8 ± 1.3 PM: NA	5	Age, sex, AL	NA
Hopf et al. (2022)^42^	Germans	H: 494 PM: 34	H: 50.2 ± 9.2 PM: 56.7 ± 9.1	H: −7.25 (median) PM: −8.75 (median)	H: NA PM: NA	5	Not tested: only 2 out of 494 eyes developed PM	Age, sex, SER, IOP

*Note*: The mean and standard deviation are used as summary statistics for baseline age, SER and AL (unless stated otherwise).

Abbreviations: AL, axial length; BL, baseline; edu, education level; Eyes, the number of eyes grouped by disease status at *baseline*; FU, follow-up duration; G/D, the development or enlargement of parapapillary gamma and delta zones; H, healthy (no pathologic myopia); IOP, baseline intraocular pressure; MM, baseline myopic maculopathy category; PF, potential prognostic factors included in the multivariable analysis; PM, pathologic myopia; RF, potential risk factors included in the multivariable analysis; SER, spherical equivalent refraction.

**TABLE 3:**
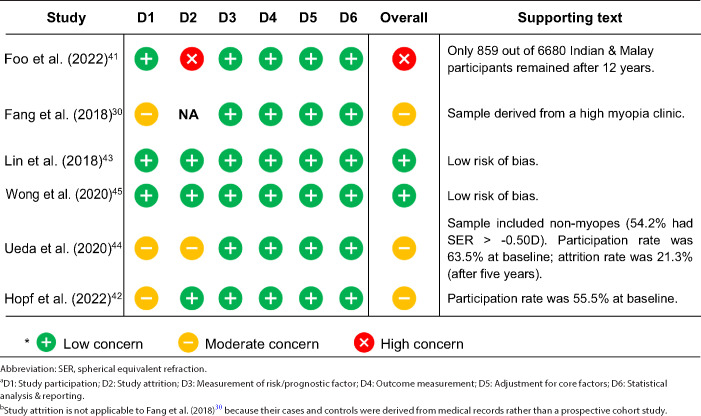
Risk of bias assessment.

Abbreviation: SER, spherical equivalent refraction.

^a^D1: Study participation; D2: Study attrition; D3: Measurement of risk/prognostic factor; D4: Outcome measurement; D5: Adjustment for core factors; D6: Statistical analysis & reporting.

^b^Study attrition is not applicable to Fang et al. (2018)^30^ because their cases and controls were derived from medical records rather than a prospective cohort study.

The incidence of PM reported by Lin et al.^43^ and Hopf et al.^42^ was only 0.05% and 0.3% over 5 years—compared with 1.1% (5 years), 1.2% (6 years), 10.3% (12 years) and 27.0% (mean ± SD: 18 ± 7 years) reported by Ueda et al.,^44^ Wong et al.,^45^ Foo et al.^41^ and Fang et al.,^30^ respectively. Due to the low incidence rates, Lin et al.^43^ and Hopf et al.^42^ could not investigate PM risk factors in their studies. The PM progression rates reported in each study were 35.3%,^43^ 50.0%,^42^ 17.0%,^45^ 12.0%^41^ and 74.3%.^30^ All studies excluded posterior staphyloma from the definition of PM and adjusted for the pre-defined core factors at the very least. The age range reported in all studies was around 30–80 years, except Fang et al.^30^ who included some children.

Considering that both Wong et al.^45^ and Foo et al.^41^ analysed the same Singapore-based SEED cohort, we included only the former study in our meta-analyses because it had a much lower risk of bias, larger sample size and was more reflective of the demographics of Singapore (data from all three main ethnic groups were available). We converted the risk ratios reported in Foo et al.^30^ and Wong et al.^45^ to ORs using the approach outlined above, using either the PM incidence or progression rate reported in these studies as baseline risk. We also added a nominal ± 0.01 to the 95% CI of the risk ratio for the prognostic factor ‘age’ in Wong et al.,^45^ as otherwise the variance would unrealistically be zero due to rounding error; that is, the reported 95% CI in that study ranged from 1.0 to 1.0, which would in turn cause the weight of the study to be undefined due to division by zero variance. We contacted the first author to get the estimates in two decimal places, but unfortunately, the raw output could not be retrieved.

### PM onset: Risk factors

**FIGURE 2:**
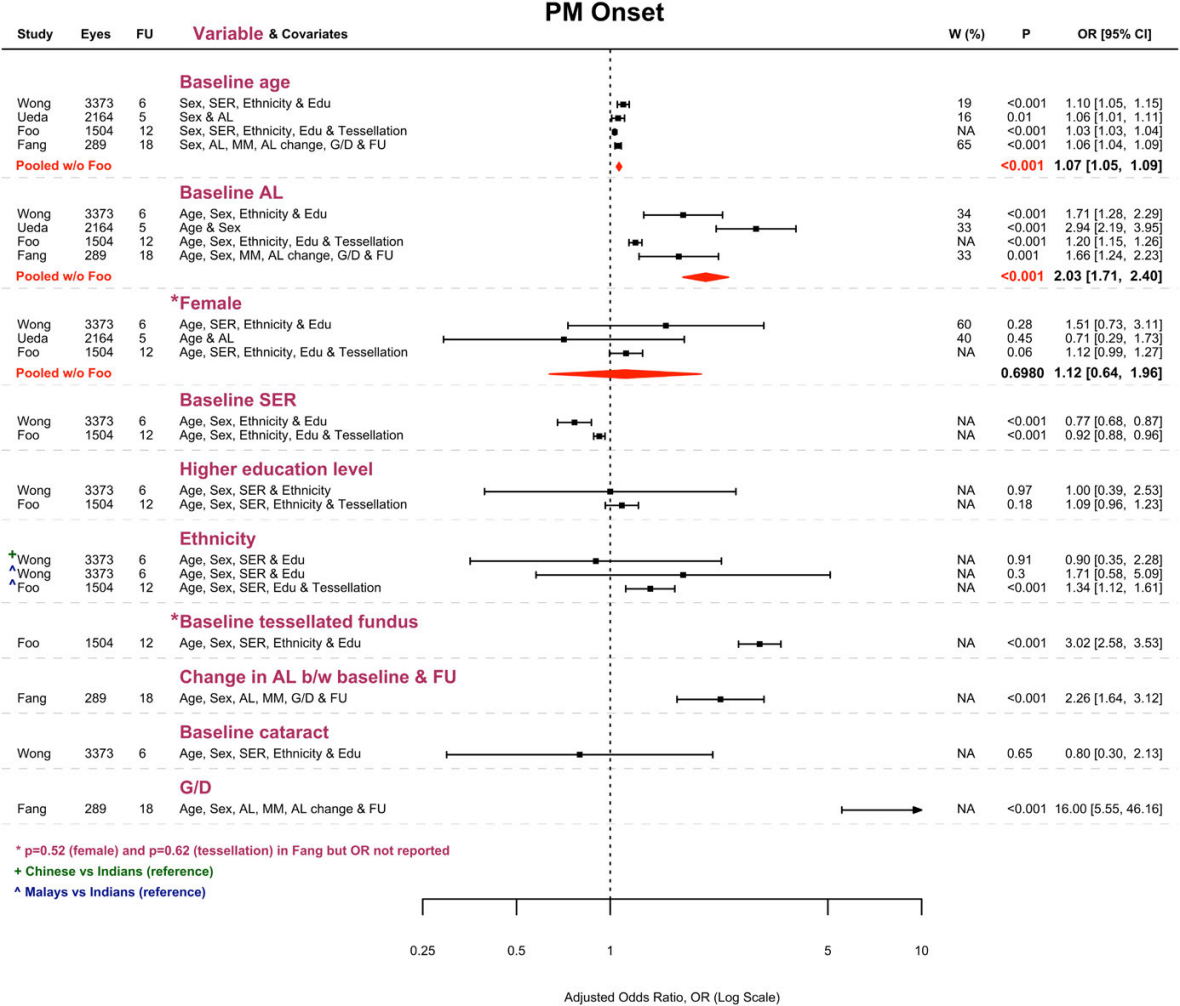
Forest plot visualising the adjusted odds ratio (OR) ± 95% CI for each potential risk factor and its pooled estimate ± 95% CI (red diamond) where meta-analysis was possible. ‘Eyes’ refers to the number of eyes without PM at baseline; ‘FU’ refers to follow-up duration (year); ‘*W* (%)’ refers to percentage study weight in meta-analysis; ‘AL’ refers to axial length; ‘SER’ refers to spherical equivalent refraction; ‘G/D’ refers to the development or enlargement of parapapillary gamma and delta zones during follow-up; ‘MM’ refers to the baseline category of myopic maculopathy. Note that ethnic Chinese, Malays and Indians from the Singapore Epidemiology of Eye Diseases cohort were included in Wong et al.,^45^ but in Foo et al.,^41^ only ethnic Malays and Indians were included. The additional papers considered here were those of Lin et al.,^43^ Hopf et al.^42^ and Fang et al.^30^

Figure 2 gives an overview of the *adjusted* OR for each risk factor explored by the included studies. Two of the variables—namely ‘G/D’ (development or enlargement of parapapillary gamma and delta zones) and ‘AL change’ (change in axial length)—were time-varying variables representing the difference between baseline and follow-up. We excluded them from evidence synthesis because the temporal relation between these factors and PM onset could not be ascertained (Bradford Hill criterion of temporality).^51^ Of the remaining baseline factors, the OR for age, axial length and sex (female as the reference level) could be meta-analysed. Older age (OR: 1.07; *p* < 0.001) and greater axial length (OR: 2.03; *p* < 0.001) were found to increase the odds of PM onset independently. In contrast, there was insufficient evidence of a difference in the odds of PM onset between sexes (OR: 1.12; *p* = 0.70). Note that Fang et al.^30^ could not be included in this meta-analysis (for sex) because they only reported the OR for statistically significant factors, but they also found no evidence of an association between female sex and the risk of PM onset (*p* = 0.52).

Of the baseline factors that were not meta-analysable, all of which were explored by studies using the Singapore-based SEED cohort unless specified otherwise, the presence of fundus tessellation at baseline appeared important because it had a large effect size (OR: 3.02; *p* < 0.001), although Fang et al.^30^ failed to find a similar association in their hospital-based sample (*p* = 0.62). A more negative SER was also found to increase the odds of PM onset over 6 years (OR: 0.77; *p* < 0.001) and 12 years (OR: 0.92; *p* < 0.001). However, a higher level of education did not appear to be a risk factor over either 6 or 12 years. Likewise, the odds of PM onset did not differ among the different ethnic groups in the SEED cohort over 6 years, although over 12 years the odds were higher in Malays than in Indians (OR: 1.34; *p* < 0.001). Finally, Wong et al.^45^ found no evidence that baseline cataract independently increased the risk of PM onset over 6 years.

### PM progression: Prognostic factors

**FIGURE 3:**
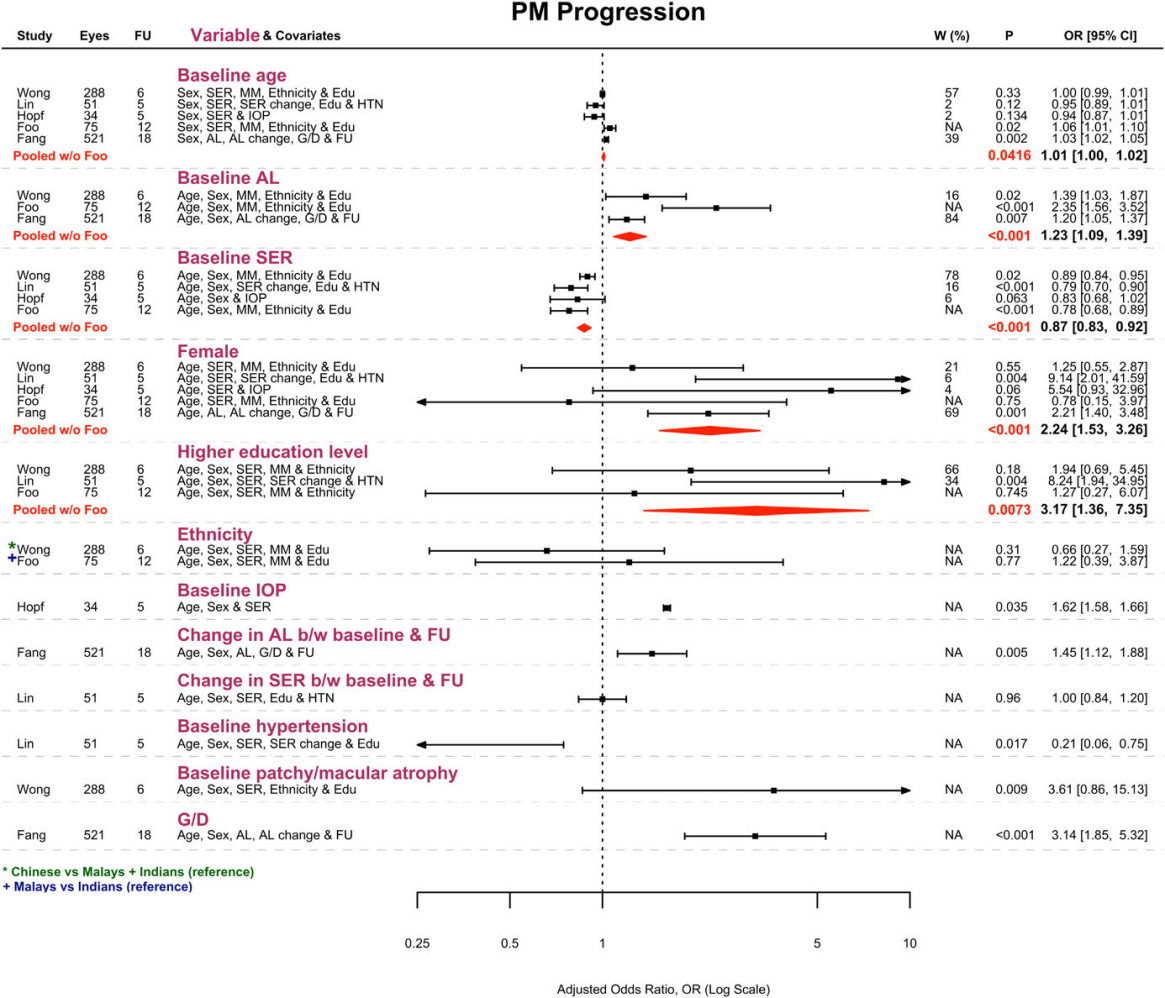
Forest plot visualising the adjusted odds ratio (OR) ± 95% CI for each potential prognostic factor and its pooled estimate ± 95% CI (red diamond) where meta-analysis was possible. ‘Eyes’ refers to the number of eyes with PM at baseline; ‘FU’ refers to follow-up duration (years); ‘*W* (%)’ refers to percentage study weight in meta-analysis; ‘MM’ refers to the baseline META-PM category of myopic maculopathy; ‘G/D’ refers to the development or enlargement of parapapillary gamma and delta zones during follow-up. Note that ethnic Chinese, Malays and Indians from the SEED cohort were included in Wong et al.,^45^ but in Foo et al.,^41^ only ethnic Malays and Indians were included. The additional papers considered here were those of Lin et al.,^43^ Hopf et al.^42^ and Fang et al.^30^

Figure 3 summarises the adjusted OR for each prognostic factor explored by the included studies. Baseline factors that could be meta-analysed included age, axial length, SER, sex and education level. Older age was found to increase the odds of PM progression (OR: 1.01; *p* = 0.04), although it should be noted that the lower limit of the 95% CI was very close to the line of null effect (OR: 1.00). Greater axial length (OR: 1.23; *p* < 0.001) and more negative SER (OR: 0.87; *p* < 0.001) were also found to increase the odds of PM progression independently. Likewise, female sex (OR: 2.24; *p* < 0.001) and higher education level (OR: 3.17; *p* < 0.01) were significantly associated with higher odds of PM progression. However, the uncertainty of these pooled risk estimates was high and heavily skewed to the right (Figure 3).

Of the baseline factors that were not meta-analysable, Hopf et al.^42^ found that higher intraocular pressure (IOP) significantly increased the odds of 5-year progression (OR: 1.62; *p* = 0.04). Conversely, Lin et al.^43^ found that hypertension reduced the odds of 5-year progression (OR: 0.21; *p* = 0.02). In the SEED cohort, Wong et al.^45^ noted that more severe myopic maculopathy at baseline (patchy chorioretinal atrophy or macular atrophy) increased the odds of 6-year progression (OR: 3.61; *p* < 0.01), although the uncertainty associated with the OR estimate was substantial (95% CI ranged from 0.86 to 15.13, crossing the line of null effect). Ethnicity did not influence the odds of progression in the SEED cohort over either 6 or 12 years.

### Sensitivity analyses

Repeating the meta-analyses using data from Foo et al.^41^ in place of Wong et al.,^45^ we similarly observed that older age (OR: 1.04; 95% CI: 1.03–1.04; *p* < 0.001) and greater axial length (OR: 1.24; 95% CI: 1.19–1.30; *p* < 0.001) increased the odds of PM onset, while the effect of female sex remained statistically insignificant (OR: 1.11; 95% CI: 0.99–1.26). Likewise, older age (OR: 1.03; 95% CI: 1.01–1.04; *p* < 0.001), greater axial length (OR: 1.28; 95% CI: 1.13–1.45; *p* < 0.001), more negative SER (OR: 0.79; 95% CI: 0.73–0.86; *p* < 0.001), female sex (OR: 2.41; 95% CI: 1.60–3.62; *p* < 0.001) and higher education level (OR: 3.48; 95% CI: 1.20–10.07; *p* = 0.02) all remained significantly associated with higher odds of PM progression.

We also investigated the impact (on the pooled OR) of adding two alternative sets of nominal values to the risk ratio reported for the prognostic (related to PM progression) factor ‘age’ in Wong et al.^45^ The first set of values was chosen to significantly bias the risk estimate towards increased risk with increasing age. Given that the reported (rounded to one decimal place) risk ratio (95% CI) corresponded to 1.0 (1.0–1.0) in the study—and that the raw 95% CI must include the null effect of 1.00 because the reported *p*-value was not significant (*p* = 0.33)—we could very conservatively assume that the risk ratio (95% CI) was 1.03 (1.00–1.04) before rounding. Repeating the meta-analysis based on this assumption, there was evidence (in agreement with the primary meta-analysis) that older age increased the odds of PM progression (OR: 1.03; 95% CI: 1.01–1.04; *p* < 0.001). The second set of values was chosen to significantly bias the risk estimate towards decreased risk with increasing age, that is, resultant risk ratio (95% CI) corresponded to 0.97 (0.96–1.00). After repeating the meta-analysis based on this assumption, however, there was no longer a significant association between age and PM progression (OR: 1.01; 95% CI: 1.00–1.02; *p* = 0.12).

### Quality of evidence

Most primary studies^30,42–45^ included in this review were exploratory in nature, which on the whole had lower quality. These studies had broad (exploratory) aims, typically looking at a wide range of factors without a clear rationale or conceptual framework, and were therefore more prone to spurious associations or could occasionally miss real effects.^52^ Foo et al.^41^ on the other hand, was the only confirmatory study because the authors explicitly sought to elucidate the independent effect of a pre-defined set of factors informed by prior studies. Table 4 presents the overall quality of evidence for each adjusted risk and prognostic factor. The rationale for the overall quality rating for each factor is detailed in Appendix S5.

**TABLE 4:**
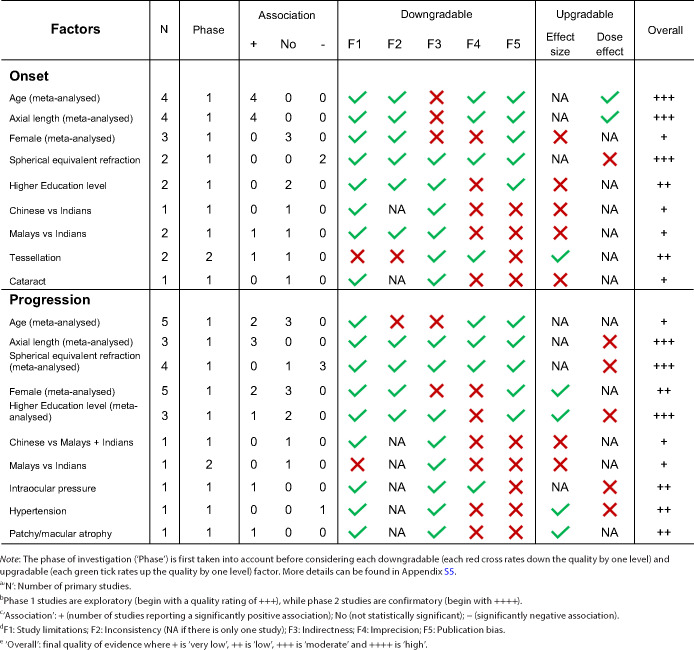
Quality of evidence for each risk and prognostic factor based on the modified Grades of Recommendation, Assessment, Development and Evaluation framework.

*Note*: The phase of investigation (‘Phase’) is first taken into account before considering each downgradable (each red cross rates down the quality by one level) and upgradable (each green tick rates up the quality by one level) factor. More details can be found in Appendix S5.

^a^‘N’: Number of primary studies.

^b^Phase 1 studies are exploratory (begin with a quality rating of +++), while phase 2 studies are confirmatory (begin with ++++).

^c^‘Association’: + (number of studies reporting a significantly positive association); No (not statistically significant); − (significantly negative association).

^d^F1: Study limitations; F2: Inconsistency (NA if there is only one study); F3: Indirectness; F4: Imprecision; F5: Publication bias.

^e^ ‘Overall’: final quality of evidence where + is ‘very low’, ++ is ‘low’, +++ is ‘moderate’ and ++++ is ‘high’.

## DISCUSSION

The existing body of literature provides an acceptable level of evidence to suggest that adults with higher myopia and older age are more likely to experience PM *onset* over 5–18 years. There is also acceptable evidence to suggest that the odds of PM *progression* increase with higher myopia and a higher level of education. However, it remains unclear whether the latter factor has any effect on the odds of PM *onset*. Likewise, whether older age is associated with PM *progression* remains an open question, not least because our sensitivity analysis yielded insufficient evidence to support this. Our review highlights significant gaps and the absence of adequate evidence for most examined factors—including relatively well explored factors such as sex, which, despite meta-analysis suggesting that females have higher odds of PM progression, is currently supported by weak evidence due to such issues as sampling bias (in the most heavily weighted study) and a highly imprecise pooled OR (Appendix S5). We should, nonetheless, stress that an ‘absence of good evidence’ is not ‘good evidence of absence’, but a strong indication that more high-quality longitudinal studies on PM are warranted.

Other factors reviewed herein, all of which could not be meta-analysed, are at risk of publication bias on the ground that very few (longitudinal) studies explored them even during model building or factor selection.^39^ These factors are discussed in the following paragraphs. First, we have very limited knowledge about whether ethnicity, particularly between populations known to be at increased risk of myopia (East Asians) and other populations,^53^ independently influences the likelihood of PM onset/progression because this has only been explored directly using data from the Singapore-based SEED cohort. Besides, indirect investigation by subgrouping studies based on ethnicity is unfeasible as it stands because most studies are on East Asian populations. Hopf et al.,^42^ the only non-Asian longitudinal study included in this review, could not investigate risk factors for PM onset and included only a limited investigation of prognostic factors for PM progression due to a small sample size. The majority of relevant studies excluded from this review were also specific to East Asian populations,^18,19,21,54–56^ with only a few involving Caucasians from Australia^57^ and Spain.^58^ Comparisons of PM prevalence among studies from different countries (see Zou et al.^59^ for example) are also unfit for elucidating the *independent* effect of ethnicity even if their outcome definitions are similar, as the confounding effect of myopia prevalence cannot be directly accounted for (higher prevalence of myopia contributes to higher prevalence of PM).^60^

Despite the presence of a large effect size (OR: 3.02), as reported by Foo et al.,^41^ there is currently only weak evidence to suggest that fundus tessellation increases the odds of PM onset—over and above baseline myopia, age and sex—considering that the study had a high risk of attrition bias and that another study^30^ failed to find a similar independent association. Having said that, fundus tessellation is known to be associated with decreased choroidal thickness,^61^ a feature indicative of reduced choroidal perfusion, which in turn may contribute to the pathogenesis of PM.^25^ In view of this, it would be worthwhile to conduct more (confirmatory) studies to corroborate the independent association between fundus tessellation and PM onset. Similarly, whether more severe myopic maculopathy at baseline is *independently* associated with higher odds of PM progression remains an open question because longitudinal evidence is currently only available from one exploratory study—notwithstanding the affirmative conclusion made by a recent meta-analysis^62^ that failed to account for the confounding effect of baseline myopia (it remains unclear whether the baseline severity of myopic maculopathy has any *added* prognostic value, since higher myopia is associated with more severe PM). Nevertheless, further investigations would still be worthwhile because a positive correlation between baseline disease severity and disease progression is commonly observed in other chronic eye conditions such as diabetic retinopathy,^63^ age-related macular degeneration^64^ and glaucoma (in functional terms).^65^ One parsimonious explanation for such apparently common associations is the existence of a vicious circle: eyes susceptible to faster disease progression end up with more severe disease, which in turn exhibit faster progression and so forth (disease progression modelling not uncommonly assumes such an interaction).^66^

Hopf et al.,^42^ the only study that examined the independent effect of baseline IOP, found a significant positive association between IOP and PM progression, though the quality of evidence is low due to the study's exploratory nature and risk of publication bias. Corneal hysteresis (viscoelasticity) is known to decrease with increasing levels of myopia^67–69^ and IOP.^70,71^ Eyes with lower corneal hysteresis may also have lower scleral hysteresis, which in turn may render them poorer at dissipating the mechanical stress from IOP.^72^ Given that reduced biomechanical resistance of the sclera has been implicated in PM,^73^ elevated IOP could plausibly be associated with increased PM susceptibility by virtue of its correlation with reduced scleral hysteresis. An alternative theoretical framework put forth by Wang et al.^74^ points towards increased activation of scleral fibroblasts leading to increased scleral remodelling and reduced choroidal perfusion in response to elevated IOP. Given the availability of reasonable conceptual frameworks supporting an association with PM, IOP appears well suited for further confirmatory investigations.

Interestingly, systemic hypertension was found to be *protective* against 5-year PM progression by Hopf et al.,^42^ even when potential confounders like age and sex were accounted for. The quality of evidence, however, is low, and a similar association was not found by Ueda et al.^44^ (adjusted for baseline age and sex) and a cross-sectional pooled analysis of the Asian Eye Epidemiology Consortium data.^60^ The latter work, if anything, suggests that systemic hypertension is more likely to increase rather than decrease the odds of myopic maculopathy (OR: 1.3, 95% CI: 1.0–1.7; *p* = 0.06). The independent effect of other comorbidities or indicators of comorbidities like diabetes, serum cholesterol level and blood pressure has only been indirectly explored by Ueda et al.^44^ during model building (none were statistically significant). Likewise, the independent effect of modifiable lifestyle factors, including smoking, alcohol consumption and physical activity, is underexplored in the literature.

It is worth noting that the risk and prognostic factors for which we currently have an acceptable level of evidence (limited in number as presented in the first paragraph) are not modifiable in adults, with baseline myopia being the only factor consistently observed to be significantly associated with both PM onset and progression. This, along with the fact that there are currently no effective treatments for PM, except anti-vascular endothelial growth factor (VEGF) for myopic choroidal neovascularisation over the short term (long-term prognosis is still not favourable),^75–77^ has two important implications. First, the best and only evidence-based measure to mitigate the risk of PM at present, it seems, is to retard myopia development and progression during childhood or adolescence.^78^ Second, more exploratory and confirmatory longitudinal studies focusing on identifying modifiable health and lifestyle factors are warranted. Moreover, existing studies almost exclusively focused on adult populations (adult-onset PM), understandably due to the rarity of childhood-onset PM. Future studies looking at PM during late childhood or adolescence may permit elucidation of the relative importance of genetic and environmental factors in early- and late-onset PM.

Very few studies in the literature included potential imaging biomarkers derived from colour fundus photographs or optical coherence tomography scans. Alterations in the posterior segment of an axially elongated eye may in themselves—or through their correlation with myopia severity—be predictive of PM susceptibility. Readers may refer to recent reviews of imaging features associated with high myopia and PM, which include (but not limited to) choroidal thinning and tilted/rotated optic disc.^79,80^ Given the paucity of longitudinal evidence for these associations, future studies should seek to corroborate whether these features are predictive of future onset and/or progression of PM. Imaging biomarkers are valuable because they have closer biological links with diseases and are more idiosyncratic than demographic or behavioural factors, making risk stratification based on imaging features more individualised.^81^ More exploratory longitudinal studies are also needed to identify novel imaging biomarkers that can then be tested with confirmatory studies.

On this note, deep learning (DL), with its ability to automatically identify and represent predictive imaging features, is valuable insofar as it is not bounded by the limits of existing domain knowledge (i.e., does not require humans to specify predictive imaging features a priori, which are mostly elusive as it stands).^82^ By probing which parts of an image a trained model focuses on, for example, DL may be a valuable adjunct to exploratory studies as a hypothesis-generating tool. Considering that significant predictive factors like myopia severity,^83^ age^84^ and sex^84^ can be predicted by DL from fundus images alone, and that imaging biomarkers may well be predictive of future PM outcomes,^85^ harnessing the immense representation power of image-based DL^82^ may enable us to make accurate projections describing the time-course trajectory of PM in individual eyes. Alongside personalised prediction of PM risk, extending the relevance of DL-based tools in future research may further improve our understanding of the disease.

## Supplementary Information

Full search strategy S1: MEDLINE (Ovid) yielded 436 references on 27 November 2022

## Data Availability

Reproducible R code and extracted data directly supporting the findings of this work are freely available at https://github.com/fyii200/pathologicMyopiaMetaAnalysis.
