# Supplementary material for: Factors associated with pathologic myopia onset and progression: A systematic review and meta-analysis
Source: Ophthalmic Physiol Opt. 2024 Apr 2;44(5):963–76. doi: 10.1111/opo.13312 (PMC12862026; doi:10.1111/opo.13312)
Supplement: Supplementary file 1 — Full search strategy S1: MEDLINE (Ovid) yielded 436 references on 27 November 2022 [file 44402_2024_4405017_MOESM1_ESM.pdf]

Full search strategy S1: *MEDLINE (Ovid) yielded 436 references on 27 November 2022*

1. Myopia, Degenerative/ (1605)
2. Choroidal Neovascularization/ (6611)
3. Myopia/ (18951)
4. 2 and 3 (232)
5. 1 or 4 (1833)
6. (myopi* adj2 (maculopathy or (macula* adj2 degenerat*))).mp. (393)
7. (staphyloma and myopia).mp. (347)
8. (lacquer crack* and myopia).mp. (119)
9. ((fuch* adj2 spot*) and myopia).mp. (32)
10. (myopi* adj2 choroid* adj2 neovascular*).mp. (343)
11. ((tessellat* adj2 (fundus or retina*)) and myopia).mp. (42)
12. (((diffuse or patch*) adj2 (chorioretina* adj2 atroph*)) and myopia).mp. (58)
13. ((macula* adj2 atroph*) and myopia).mp. (81)
14. (pathologi* adj2 myopi*).mp. (756)
15. 5 or 6 or 7 or 8 or 9 or 10 or 11 or 12 or 13 or 14 (2529)
16. (validat* or predict* or rule*).mp. (2285051)
17. (predict* and (outcome* or risk* or model*)).mp. (1059186)
18. ((History or variable* or criteria or scor* or characteristic* or finding* or factor*) and (predict* or model* or decision* or identif* or prognos*)).mp. (4139387)
19. decision*.mp. and ((model* or clinical*).mp. or Logistic Models/) (243056)
20. ((prognos* or risk*) and (history or variable* or criteria or scor* or characteristic* or finding* or factor* or model*)).mp. (2525654)
21. Risk Factors/ or (risk adj2 factor*).mp. (1231948)
22. 16 or 17 or 18 or 19 or 20 or 21 (6312175)
23. 15 and 22 (779)
24. exp animals/ not humans.sh. (5063870)
25. 23 not 24 (768)
26. limit 25 to english language (729)
27. limit 26 to yr="2015 -Current" (436)

Full search strategy S2: *EMBASE (Ovid) yielded 531 references on 27 November 2022*

1. exp degenerative myopia/ (405)
2. (myopi* adj2 (maculopathy or (macula* adj2 degenerat*))).mp. (610)
3. (staphyloma and myopia).mp. (585)
4. (lacquer crack* and myopia).mp. (166)
5. ((fuch* adj2 spot*) and myopia).mp. (52)
6. (myopi* adj2 choroid* adj2 neovascular*).mp. (530)
7. ((tessellat* adj2 (fundus or retina*)) and myopia).mp. (74)
8. (((diffuse or patch*) adj2 (chorioretina* adj2 atroph*)) and myopia).mp. (80)
9. ((macula* adj2 atroph*) and myopia).mp. (127)
10. (pathologi* adj2 myopi*).mp. (1198)
11. 1 or 2 or 3 or 4 or 5 or 6 or 7 or 8 or 9 or 10 (2693)
12. (validat* or predict* or rule*).mp. (3711444)
13. (predict* and (outcome* or risk* or model*)).mp. (1714646)
14. ((History or variable* or criteria or scor* or characteristic* or finding* or factor*) and (predict* or model* or decision* or identif* or prognos*)).mp. (6387307)
15. decision*.mp. and ((model* or clinical*).mp. or Logistic Models/) (547834)
16. ((prognos* or risk*) and (history or variable* or criteria or scor* or characteristic* or finding* or factor* or model*)).mp. (4031825)
17. Risk Factors/ or (risk adj2 factor*).mp. (1667682)
18. 12 or 13 or 14 or 15 or 16 or 17 (9917417)
19. 11 and 18 (846)
20. exp animal/ not human/ (5177972)
21. 19 not 20 (829)
22. limit 21 to english language (769)
23. limit 22 to yr="2015 -Current" (531)

Full search strategy S3: *SCOPUS yielded 472 references on 27 November 2022*

(TITLE-ABS-KEY (degenerative AND myopia) OR TITLE-ABS-KEY (myopi* W/1 (maculopathy OR (macula* W/1 degenerat*))) OR TITLE-ABS-KEY (staphyloma AND myopia) OR TITLE-ABS-KEY (lacquer AND crack* AND myopia) OR TITLE-ABS-KEY ((fuch* W/1 spot*) AND myopia) OR TITLE-ABS-KEY (myopi* W/1 choroid* W/1 neovascular*) OR TITLE-ABS-KEY ((tessellat* W/1 (fundus OR retina*)) AND myopia) OR TITLE-ABS-KEY (((diffuse OR patch*) W/1 (chorioretina* W/1 atroph*)) AND myopia) OR TITLE-ABS-KEY ((macula* W/1 atroph*) AND myopia) OR TITLE-ABS-KEY (pathologi* W/1 myopi*)) AND (TITLE-ABS-KEY (risk OR prognos* OR factor* OR predict* OR model* OR feature* OR characteristic*) AND TITLE-ABS-KEY (onset OR develop* OR progress* OR worsen* OR improve*)) AND PUBYEAR > 2014 AND PUBYEAR < 2023 AND (EXCLUDE (DOCTYPE , "re") OR EXCLUDE (DOCTYPE , "le") OR EXCLUDE (DOCTYPE , "sh") OR EXCLUDE (DOCTYPE , "no") OR EXCLUDE (DOCTYPE , "ed")) AND (LIMIT-TO (LANGUAGE , "English"))

S4: *Risk of bias domains and signaling items based on the Quality in Prognosis Studies (QUIPS) framework.*[1]

Domains	Signaling Items	Risk of Bias Ratings
1. Study participation	(a) Adequate participation in the study by eligible persons<br><br>(b) Description of the target population or population of interest<br><br>(c) Description of the baseline study sample<br><br>(d) Adequate description of the sampling frame and recruitment<br><br>(e) Adequate description of the period and place of recruitment<br><br>(f) Adequate description of inclusion and exclusion criteria	**High:** *the relationship between the risk/prognostic factor and outcome is very likely to be different for participants and eligible non-participants*<br><br>**Moderate:** *the relationship between the risk/prognostic factor and outcome may be different for participants and eligible non-participants*<br><br>**Low:** *the relationship between the risk/prognostic factor and outcome is unlikely to be different for participants and eligible non-participants*
2. Study attrition	(a) Adequate participation rate and low attrition rate<br><br>(b) Description of attempts to collect information on participants who dropped out<br><br>(c) Reasons for loss to follow-up are provided<br><br>(d) Adequate description of participants lost to follow-up<br><br>(e) There are no important differences between participants who completed the study and those who did not	**High:** *the relationship between the risk/prognostic factor and outcome is very likely to be different for completing and non-completing participants*<br><br>**Moderate:** *the relationship between the risk/prognostic factor and outcome may be different for completing and non-completing participants*<br><br>**Low:** *the relationship between the risk/prognostic factor and outcome is unlikely to be different for completing and non-completing participants*
3. Risk/prognostic factor measurement	(a) A clear definition or description of the factor is provided<br><br>(b) Method of factor measurement is adequately valid and reliable<br><br>(c) Continuous variables are reported or appropriate cut-points (i.e., not data-dependent) are used<br><br>(d) The method and setting of measurement of factor is the same for all study participants<br><br>(e) Adequate proportion of the study sample has complete data for the factor<br><br>(f) Appropriate methods of imputation are used for missing data	**High**: *the measurement of the risk/prognostic factor is very likely to be different for different levels of the outcome of interest*<br><br>**Moderate**: *the measurement of the risk/prognostic factor may be different for different levels of the outcome of interest*<br><br>**Low**: *the measurement of the risk/prognostic factor is unlikely to be different for different levels of the outcome of interest*
4. Outcome measurement	(a) A clear definition of the outcome is provided<br><br>(b) Method of outcome measurement used is adequately valid and reliable	**High**: *the measurement of the outcome is very likely to be different across different baseline levels of the risk/prognostic factor*<br><br>**Moderate**: *the measurement of the*

	(c) The method and setting of outcome measurement is the same for all study participants	*outcome may be different across different baseline levels of the risk/prognostic factor*<br><br>**Low**: *the measurement of the outcome is unlikely to be different across different baseline levels of the risk/prognostic factor*
5. Inappropriate adjustment for or measurement of core factors	(a) All core factors are measured, i.e., baseline severity of myopia measured either as AL or SER, baseline age and sex<br><br>(b) Measurement of all core factors is adequately valid and reliable<br><br>(c) The method and setting of core factor measurement are the same for all study participants<br><br>(d) Appropriate methods are used to deal with missing values of core factors, such as multiple imputation<br><br>(e) Core factors are accounted for in the analysis	**High**: *the observed effect of the risk/prognostic factor on the outcome is very likely to be distorted by how core factors are measured*<br><br>**Moderate**: *the observed effect of the risk/prognostic factor on the outcome may be distorted by how core factors are measured*<br><br>**Low**: *the observed effect of the risk/prognostic factor on the outcome is unlikely to be distorted by how core factors are measured*
6. Statistical analysis and reporting	(a) Sufficient presentation of data to assess the adequacy of the analytic strategy<br><br>(b) Strategy for model building is appropriate<br><br>(c) The selected statistical model is adequate for the design of the study<br><br>(d) There is no selective reporting of results	**High**: *the reported results are very likely to be spurious or biased*<br><br>**Moderate**: *the reported results may be spurious or biased*<br><br>**Low**: *the reported results are unlikely to be spurious or biased*

S5: *Quality of evidence rating based on an adapted version of the Grades of Recommendation, Assessment, Development and Evaluation (GRADES) framework.*[2]

- Overall quality rating (ordinal scale): “Very low”, “Low”, “Moderate” & “High”.

- The initial quality rating was determined by the phase of investigation[3] of the primary study/studies (“high” if the study was confirmatory and “moderate” if exploratory. All studies except Foo et al.[4] were **exploratory** in nature. Lin et al.[5] explored factors as diverse as height, weight, diabetes, smoking, alcohol consumption, hypertension, age, sex, education level, SER and change in SER using univariable logistic regression before including statistically significant factors (level of significance, α=0.1) in their final model. Ueda et al.[6] used a backward elimination approach in that nonsignificant factors (α=0.1) were discarded from their initial full model that included height, body mass index, systolic blood pressure, diastolic blood pressure, hypertension, diabetes, serum total cholesterol, smoking habits, alcohol intake, regular exercise, age, sex and AL. Wong et al.[7] also employed backward elimination to select factors from a diverse pool of variables including disc lesions, cataract, glaucoma, diabetic retinopathy, age-related macular degeneration, SER, AL, education level, ethnicity, age and sex. Hopf et al.[8] did not detail their factor selection process, including the rationale for including IOP, so the study was judged to be exploratory in nature. Fang et al.[9] explored factors including sex, AL, best-corrected visual acuity, change in AL, follow-up duration and G/D using backward elimination. Foo et al.[4] was a **confirmatory** study because it explicitly sought to investigate the independent effect of a pre-defined set of factors based on previous research.

- For factors that were meta-analysed, the default initial rating was “moderate” because Foo et al.,[4] which was the only confirmatory study, was not included in any primary meta-analysis.

- From the initial quality rating, the evidence was downgraded each time there was evidence of a serious concern pertaining to **study limitation** (high risk of bias based on the QUIPS assessment), **inconsistency** (inconsistent effect directions across primary studies with minimal to no overlap of 95% CIs and/or inconsistent results between primary meta-analysis and sensitivity analysis), **indirectness** (study sample not matching our targeted population), **imprecision** (a large spread of 95% CI indicating that the estimate of OR is highly uncertain) or **publication bias** (default position is to assume that the evidence for any given risk or prognostic factor is seriously affected by publication bias unless the factor concerned has been explored by several cohort studies).[2] Note that in addition to age, sex and myopia severity, education level as a potential risk/prognostic factor is well explored by studies included[4, 5, 7] and excluded[10-12] from the present review, so it’s not judged to be at a high risk of publication bias.

- The quality rating was upgraded each time there was sufficient evidence of a **dose effect** (dose-response gradient; applicable to continuous factors only) or **large effect size** (OR>2; applicable to categorical factors only)

- Only baseline factors were considered (i.e., factors related to changes between baseline and follow-up were excluded from both narrative and quantitative synthesis).

PM onset

Potential risk factors	Overall quality	Rationale
**Age (meta-analysed)**	Moderate	From "moderate", the quality of evidence was downgraded once due to the inclusion of non-myopes in Ueda et al.[6] and the use of a hospital-based sample in Fang et al.[9] resulted in some indirectness of evidence. This quality was, however, upgraded once due to the presence of a clear dose-dependent effect (Figure 1 in Ueda et al.[6]).
**Axial length (meta-analysed)**	Moderate	Same as "Age". PS: dose-dependent effect is evident from Figure 2 in Ueda et al.[6]
**Female (meta-analysed)**	Very low	From "moderate", the quality of evidence was downgraded twice due to: (1) inclusion of non-myopes in Ueda et al.[6], which was a study with considerable influence on the pooled estimate (40%), resulting in some indirectness of evidence; and (2) highly imprecise point estimate characterised by a wide 95% CI, particularly in the two largest (meta-analysed) studies. PS: although there was some inconsistency in effect directions between Ueda et al.[6] (pointed towards reduced odds) and studies using the Singapore-based SEED cohort[4, 7] (pointed towards increased odds), we did not rate down the quality for inconsistency because their 95% CIs showed sufficient overlap.
**Spherical equivalent refraction**	Moderate	Evidence was narratively synthesised from two studies that analysed the same cohort over different follow-up periods (6 years in Wong et al.[7] and 12 years in Foo et al.[4]). More weight was given to Wong et al.[7] because of its lower risk of bias (Foo et al.[4] had a very high attrition rate) and larger sample size. As such, the quality of evidence started at "moderate" (Wong et al.[7] was exploratory rather than confirmatory). From here, there were neither downgradable nor upgradable points, leading to a final rating of "moderate".
**Higher education level**	Low	Same as "spherical equivalent refraction": evidence was narratively synthesised from both Wong et al.[7] (given greater emphasis due to its larger sample size and lower risk of bias) and Foo et al.[4] From "moderate", the quality of evidence was downgraded once due to the highly imprecise point estimate in Wong et al.[7] (95% CI: 0.39 to 2.53).
**Chinese vs Indians**	Very low	From "moderate", the quality of evidence was downgraded twice due to: (1) highly imprecise point estimate (95% CI: 0.35 to 2.28); and (2) risk of publication bias (Chinese ethnicity as an independent factor has only been explored by Wong et al.[7]).
**Malays vs Indians**	Very low	Evidence was narratively synthesised from two studies (Wong et al.[7] and Foo et al.[4]). Greater emphasis was given to Wong et al.[7] due to the same reasons as before. From "moderate", the quality of evidence was downgraded twice due to: (1) highly imprecise point estimate in Wong et al.[7] (95% CI: 0.58 to 5.09); and (2) risk of publication bias (as explained earlier).
**Tessellation**	Low	Evidence was narratively synthesised from two studies (Foo et al.[4] and Fang et al.[9]). Foo et al.[4] was the only study that reported the OR ± 95% CI, so it was given more emphasis. The quality rating started at "high" (as Foo et al.[4] was a confirmatory study) but was downgraded three times due to: (1) high risk of bias arising from a high attrition rate; (2) inconsistent findings between Foo et al.[4] (significant association) and Fang et al.[9] (no significant association); and (3) risk of publication bias. The quality was, however, upgraded once on account of its large effect size with a relatively precise point estimate (OR: 3.02).
**Cataract**	Very low	From "moderate", the quality of evidence was downgraded twice due to: (1) imprecise point estimate (95% CI: 0.30 to 2.13); and (2) risk of publication bias (only explored by Wong et al.[7]).

PM progression

Potential prognostic factors	Overall quality	Rationale
**Age (meta-analysed)**	Very low	From "moderate", the quality of evidence was downgraded twice due to: (1) inconsistent effect directions across primary studies with minimal overlap of 95% CIs (2 studies[5, 8] pointed towards reduced odds with older age, while two other[4, 9] pointed towards higher odds with older age), and contradictory results between the primary meta-analysis (significant positive association) and sensitivity analysis (no significant association); and (2) indirectness of evidence because Fang et al.,[9] being the largest study and the only large study that reported increased odds of progression with older age, used a hospital-based (rather than population-based) sample. Thus, we could not rule out the possibility that the significant positive association between age and progression odds as indicated by our meta-analysis was not influenced by this source of bias.
**Axial length (meta-analysed)**	Moderate	From "moderate", there were neither downgradable nor upgradable points. Note that although the most heavily weighted study (Fang et al.[9]) derived their sample from a hospital-based population (indirectness of evidence), we did not rate down the quality of evidence for indirectness because Wong et al.[7], which had a large sample size but **did not** have the issue of indirectness, **similarly** suggested higher progression odds with greater AL. Foo et al.[4] (no issue of indirectness) also found a similar association. We were, therefore, quite certain that the positive association reported by Fang et al.[9] could not have been caused by this source of bias.
**Spherical equivalent refraction (meta-analysed)**	Moderate	From "moderate", there were neither downgradable nor upgradable points.
**Female (meta-analysed)**	Low	From "moderate", the quality of evidence was downgraded twice due to: (1) the use of a hospital-based sample by Fang et al.[9] (indirectness of evidence), which was the most heavily weighted study (69%); and (2) highly imprecise point estimate (pooled 95% CI: 1.53 to 3.26). The quality was rated up once due to the presence of a large effect size (OR: 2.24 or 124% increase in odds in females). PS: although the point estimates in Foo et al.[4] (pointed towards reduced odds) and other studies[5, 7-9] (pointed towards increased odds) did not point in the same direction, we did not rate down the quality for inconsistency because there was a significant overlap of their 95% CIs.
**Higher education level (meta-analysed)**	Moderate	From "moderate", the quality of evidence was downgraded once because the pooled estimate was highly imprecise (95% CI: 1.36 to 7.35), but this was offset (i.e., quality rated up once) by the presence of a large effect size (pooled OR was 3.17), which increased our certainty that there's *at least* some effect.
**Chinese vs Malays + Indians (reference)**	Very low	From "moderate", the quality of evidence was downgraded twice due to: (1) imprecise point estimate (95% CI: 0.27 to 1.59); and (2) risk of publication bias (Chinese ethnicity as an independent factor has only been explored by one study).
**Malays vs Indians (reference)**	Very low	Evidence was synthesised from a confirmatory study (Foo et al.[4]), so the quality rating started at "high", but this was downgraded three times due to: (1) high risk of bias arising from a high attrition rate (significant study limitation); (2) highly imprecise point estimate (95% CI: 0.39 to 3.87); (3) risk of publication bias (only explored by one study).
**Intraocular pressure**	Low	From "moderate", the quality of evidence was downgraded once due to a high risk of publication bias, considering that IOP was only explored by one study and the rationale was not given.
**Hypertension**	Low	From "moderate", the quality of evidence was downgraded twice due to: (1) highly imprecise point estimate (95% CI with presence of hypertension as reference: 1÷0.75=**1.33 to** 1÷0.06=**16.67**); and (2) risk of publication bias (only considered by two included studies, both of which were exploratory). The quality, however, was upgraded once due to a large effect size (OR with presence of hypertension as reference: 1÷0.21=**4.76** or 376% increase in odds in the absence of hypertension).
**Patchy/macular atrophy**	Low	From "moderate", the quality of evidence was downgraded twice due to: (1) highly imprecise OR estimate which also crossed the line of null effect (95% CI: 0.86 to 15.13); and (2) high risk of publication bias, as it was only explored by one included study.[7] The quality was, however, upgraded once due to a large effect size (OR: 3.14).

References

1. Hayden JA, van der Windt DA, Cartwright JL, Côté P, Bombardier C. Assessing bias in studies of prognostic factors. Ann Intern Med. 2013;158(4):280-6.
2. Huguet A, Hayden JA, Stinson J, McGrath PJ, Chambers CT, Tougas ME, et al. Judging the quality of evidence in reviews of prognostic factor research: Adapting the GRADE framework. Syst Rev. 2013;2:71. Epub 20130905.
3. Hayden JA, Côté P, Steenstra IA, Bombardier C, Group Q-LW. Identifying phases of investigation helps planning, appraising, and applying the results of explanatory prognosis studies. J Clin Epidemiol. 2008;61(6):552-60. Epub 20080310.
4. Foo LL, Xu L, Sabanayagam C, Htoon HM, Ang M, Zhang J, et al. Predictors of myopic macular degeneration in a 12-year longitudinal study of Singapore adults with myopia. Br J Ophthalmol. 2022. Epub 20220509.
5. Lin C, Li SM, Ohno-Matsui K, Wang BS, Fang YX, Cao K, et al. Five-year incidence and progression of myopic maculopathy in a rural Chinese adult population: The Handan Eye Study. Ophthalmic Physiol Opt. 2018;38(3):337-45.
6. Ueda E, Yasuda M, Fujiwara K, Hashimoto S, Ohno-Matsui K, Hata J, et al. Five-year incidence of myopic maculopathy in a general Japanese population: The Hisayama Study. JAMA Ophthalmol. 2020;138(8):887-93.
7. Wong YL, Sabanayagam C, Wong CW, Cheung YB, Man REK, Yeo AC, et al. Six-year changes in myopic macular degeneration in adults of the Singapore Epidemiology of Eye Diseases Study. Invest Ophthalmol Vis Sci. 2020;61(4):14.
8. Hopf S, Heidt F, Korb CA, Schulz A, Münzel T, Wild PS, et al. Five-year cumulative incidence and progression of myopic maculopathy in a German population. Ophthalmology. 2022;129(5):562-70. Epub 20211227.
9. Fang Y, Yokoi T, Nagaoka N, Shinohara K, Onishi Y, Ishida T, et al. Progression of myopic maculopathy during 18-year follow-up. Ophthalmology. 2018;125(6):863-77. Epub 20180119.
10. Chen SJ, Cheng CY, Li AF, Peng KL, Chou P, Chiou SH, et al. Prevalence and associated risk factors of myopic maculopathy in elderly Chinese: the Shihpai eye study. Invest Ophthalmol Vis Sci. 2012;53(8):4868-73. Epub 20120724.
11. Liu HH, Xu L, Wang YX, Wang S, You QS, Jonas JB. Prevalence and progression of myopic retinopathy in Chinese adults: The Beijing Eye Study. Ophthalmology. 2010;117(9):1763-8. Epub 20100505.
12. Wong YL, Zhu X, Tham YC, Yam JCS, Zhang K, Sabanayagam C, et al. Prevalence and predictors of myopic macular degeneration among Asian adults: Pooled analysis from the Asian Eye Epidemiology Consortium. Br J Ophthalmol. 2021;105(8):1140-8. Epub 20200902.
